# Regional citrate anticoagulation in CVVH: A new protocol combining citrate solution with a phosphate-containing replacement fluid

**DOI:** 10.1111/j.1542-4758.2012.00730.x

**Published:** 2012-08-07

**Authors:** Santo Morabito, Valentina Pistolesi, Luigi Tritapepe, Laura Zeppilli, Francesca Polistena, Enrico Fiaccadori, Alessandro Pierucci

**Affiliations:** 1 Department of Nephrology and Urology, Hemodialysis Unit, Umberto I, Policlinico di Roma, “Sapienza” UniversityRome, Italy; 2 Department of Anesthesiology and Intensive Care, Cardiac Surgery ICU, Umberto I, Policlinico di Roma, “Sapienza” UniversityRome, Italy; 3 Department of Internal Medicine, Nephrology and Health Sciences, University of Parma Medical SchoolParma, Italy

**Keywords:** Acute kidney injury, CVVH, citrate, regional citrate anticoagulation, phosphate, hypophosphatemia

## Abstract

Regional citrate anticoagulation (RCA) is a valid anticoagulation method in continuous renal replacement therapies (CRRT) and different combination of citrate and CRRT solutions can affect acid-base balance. Regardless of the anticoagulation protocol, hypophosphatemia occurs frequently in CRRT. In this case report, we evaluated safety and effects on acid-base balance of a new RCA- continuous veno-venous hemofiltration (CVVH) protocol using an 18 mmol/L citrate solution combined with a phosphate-containing replacement fluid. In our center, RCA-CVVH is routinely performed with a 12 mmol/L citrate solution and a postdilution replacement fluid with bicarbonate (protocol A). In case of persistent acidosis, not related to citrate accumulation, bicarbonate infusion is scheduled. In order to optimize buffers balance, a new protocol has been designed using recently introduced solutions: 18 mmol/L citrate solution, phosphate-containing postdilution replacement fluid with bicarbonate (protocol B). In a cardiac surgery patient with acute kidney injury, acid-base status and electrolytes have been evaluated comparing protocol A (five circuits, 301 hours) vs. protocol B (two circuits, 97 hours): pH 7.39 ± 0.03 vs. 7.44 ± 0.03 (P < 0.0001), bicarbonate 22.3 ± 1.8 vs. 22.6 ± 1.4 mmol/L (NS), Base excess −2.8 ± 2.1 vs. −1.6 ± 1.2 (P = 0.007), phosphate 0.85 ± 0.2 vs. 1.3 ± 0.5 mmol/L (P = 0.027). Protocol A required bicarbonate and sodium phosphate infusion (8.9 ± 2.8 mmol/h and 5 g/day, respectively) while protocol B allowed to stop both supplementations. In comparison to protocol A, protocol B allowed to adequately control acid-base status without additional bicarbonate infusion and in absence of alkalosis, despite the use of a standard bicarbonate concentration replacement solution. Furthermore, the combination of a phosphate-containing replacement fluid appeared effective to prevent hypophosphatemia.

## Introduction

Continuous renal replacement therapies (CRRT) are widely adopted in the management of severe acute kidney injury (AKI) in critically ill patients with hemodynamic instability and multiple organ dysfunction syndrome.^1^–^3^ A potential drawback of CRRT is the need for prolonged anticoagulation to prevent clotting of the extracorporeal circuit.^4^ The risk of bleeding and/or the development of heparin-induced thrombocytopenia contributed to an increasing interest in the use of alternative strategies.^5^–^9^ Among them, regional citrate anticoagulation (RCA) seems to be a valid option in patients at high risk of bleeding.^7^,^8^,^10^ Since citrate is a small molecule (MW 294 Da), calcium-citrate complex is easily removed by diffusion and/or convection and systemic calcium infusion is thus required to replace the calcium lost in the effluent.^11^ The citrate metabolic load derives from the balance between citrate dose and the amount removed by filtration and/or dialysis.^11^ Citrate returning to the patient is rapidly metabolized to bicarbonate mainly in the liver, but also in skeletal muscle and renal cortex.^7^ Reported derangements with RCA include metabolic alkalosis and acidosis, hyper- and hyponatremia, hypocalcemia, but these complications are uncommon if an accurate monitoring is made.^7^,^12^ Reported RCA protocols are characterized by variability in CRRT modality, citrate metabolic load and composition of citrate and CRRT solutions, in many cases customized and hospital pharmacy formulated.^13^ However, the availability of dedicated commercial solutions could contribute to simplify protocols allowing to improve safety and to extend the use of RCA. Different combinations of citrate and CRRT solutions, as well as variation of operational parameters setting, can be associated with a high variability of buffers supply, significantly affecting the acid-base status. Regardless of the anticoagulation protocol, hypophosphatemia occurs frequently in CRRT (10–80%).^14^–^19^ Indeed, the conventional solutions adopted in CRRT do not contain phosphate and the incidence of hypophosphatemia is increased by the use of high dialysis dose and during prolonged treatment.^15^,^16^,^18^

In this case report, we evaluated the safety and effects on electrolyte and acid-base status of a new RCA protocol in CRRT using an 18 mmol/L citrate solution combined with a phosphate-containing replacement solution.

## Case Report

**Figure 1:**
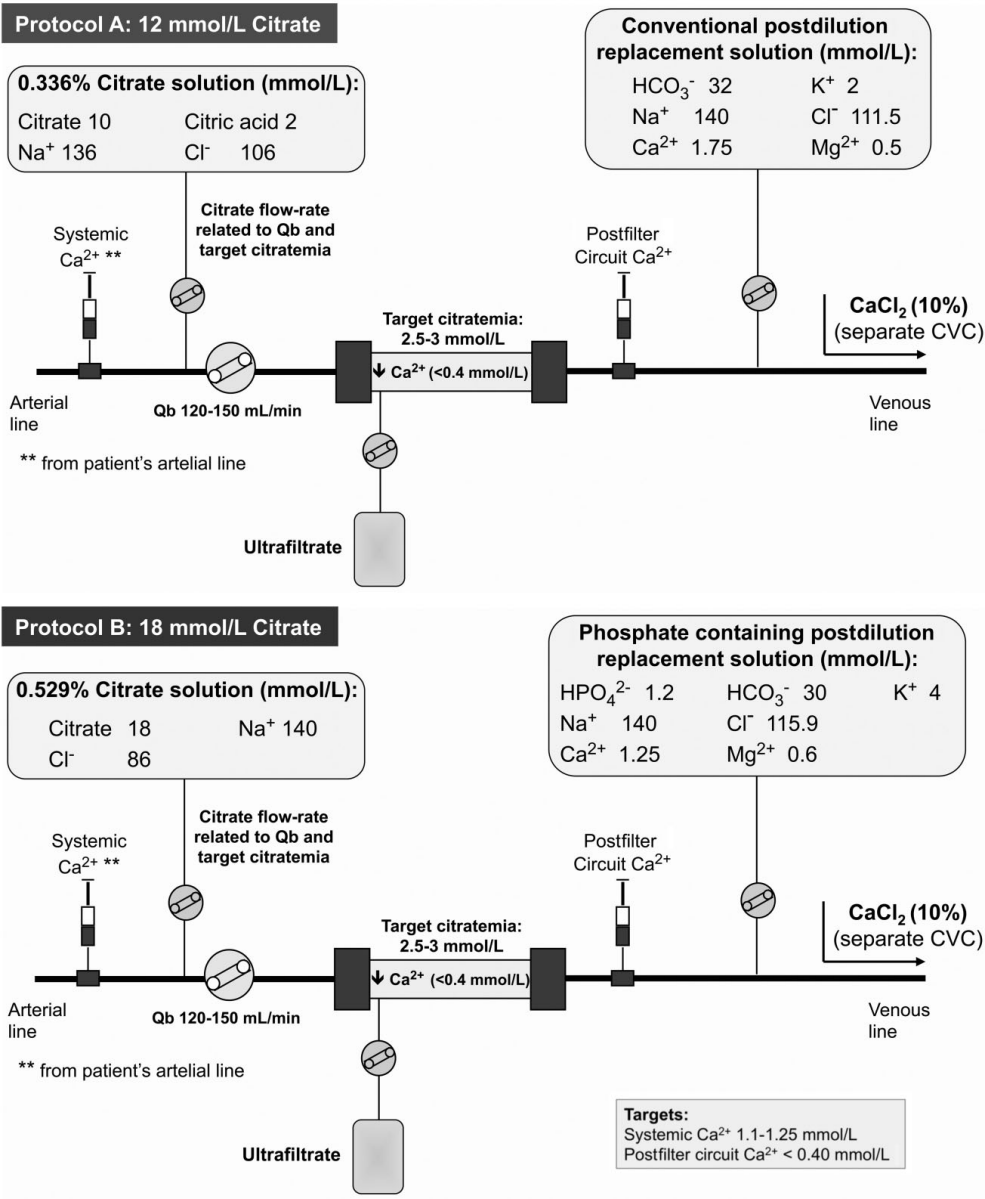
Regional citrate anticoagulation protocols in pre–postdilution CVVH modality.

On September 2011, a 78-year-old Caucasian woman (body weight 60 kg, Sequential Organ Failure Assessment score 16, Acute Physiology and Chronic Health Evaluation II score 35) with acute myocardial infarction was admitted to cardiac surgery intensive care unit (ICU) after coronary artery bypass graft combined with mitral valve plastic. According to the American College of Chest Physicians Evidence-Based Clinical Practice guidelines,^20^ after the admission in postoperative ICU, deep venous thrombosis prophylaxis was started (calcium heparin 5000 IU every 8 hours). Because of hemodynamic instability, unresponsive to high dose inotropic and vasopressor support (cardiovascular SOFA 4), intra-aortic balloon pump counterpulsation was started and maintained for the next 72 hours. At the same time, the patient developed oliguria and severe AKI requiring renal replacement therapy. Informed consent for the treatment has been obtained from patient's close relative. Because of thrombocytopenia (PLT 45000/μL), CRRT was started without anticoagulation. No anticoagulation CRRT was associated with early filter failure (< 24 hours); therefore, the treatment was switched to RCA. In our center, RCA is routinely performed in continuous veno-venous hemofiltration modality (RCA-CVVH) with a 12 mmol/L predilution citrate solution (Prismocitrate 10/2, Gambro, Sondalo, Italy) and a postdilution replacement solution with bicarbonate (Prismasol 2, Gambro) (protocol A; Figure 1). In the case of metabolic acidosis, not related to citrate accumulation and persisting after RCA-CVVH parameters optimization, additional bicarbonate infusion in a separate line is scheduled.

Starting from September 2011, a new protocol (protocol B) has been designed in order to optimize buffers balance and to reduce the need for phosphate and potassium supplementation, throughout the adoption of the following recently introduced solutions: 18 mmol/L predilution citrate solution (Prismocitrate 18/0, Gambro) combined with phosphate-containing postdilution replacement fluid (Phoxilium, Gambro; Figure 1). The protocol has been designed throughout a mathematical model developed to roughly estimate citrate and bicarbonate mass transfer as well as main RCA-CVVH parameters. The model, included in a database software (FileMaker Inc., Santa Clara, CA, USA), compatible with many portable devices, allowed to easily make calculations bedside. RCA-CVVH was performed using the Prismaflex system (Gambro Lundia AB, Lund, Sweden) and PAES hemofilters (HF 1000, 1.15 m^2^, Gambro, Meyzieu, France). In relation to blood flow rate (Qb), the citrate solution rate was set to meet the target circuit citrate concentration of 2.5–3 mmol/L (calculated in plasma water)^21^,^22^ and modified, if needed, to obtain circuit Ca^2+^ (c-Ca^2+^) ≤ 0.4 mmol/L. Postdilution bicarbonate solution rate (Prismasol 2 for protocol A, Phoxilium for protocol B) was adjusted to achieve a prescribed dialysis dose, corrected for predilution, of about 30 mL/kg/h with the aim to ensure a delivered dialysis dose of at least 25 mL/kg/h. Calcium chloride (10%) was infused in a separate line to maintain target systemic Ca^2+^ (s-Ca^2+^) (1.1-1.25 mmol/L), measured by arterial blood gases, as well as potassium, at least every 4 hours. A total calcium/Ca^2+^ ratio > 2.5 was considered an indirect sign of citrate accumulation. Serum electrolytes, including Ca, P, Mg, coagulation parameters and complete blood count were daily assessed. Potassium, P and Mg loss with CVVH was replaced, if needed, with potassium chloride, sodium phosphate and magnesium sulfate supplementation.

**Figure 2:**
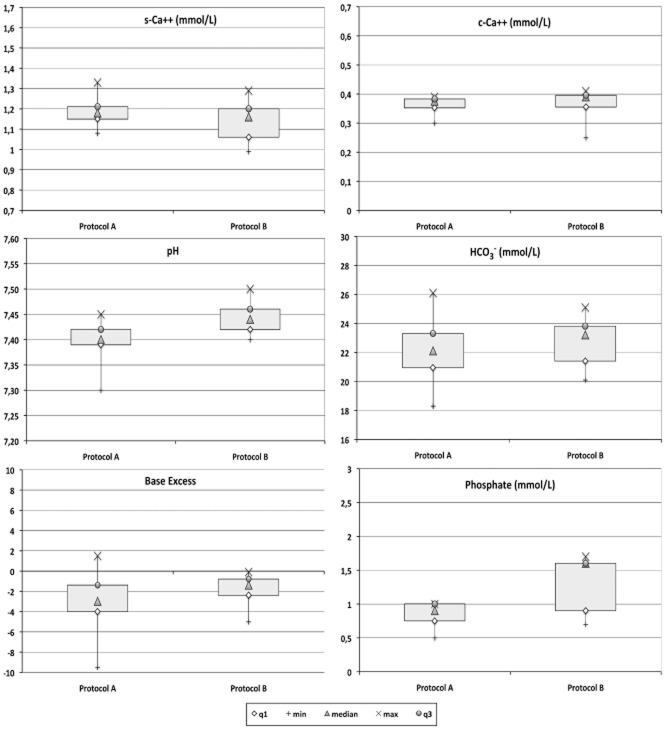
Systemic and circuit Ca^2+^, as well as acid-base parameters and phosphate during protocol A and protocol B periods. Data are expressed as median, interquartile range (q1 to q3), minimum (min), maximum (max).

Acid-base status and serum electrolytes have been evaluated in this patient comparing protocol A (five circuits, total running time 301 hours) versus protocol B (two circuits, total running time 97 hours). RCA-CVVH was never stopped for clotting events. Systemic Ca^2+^ and c-Ca^2+^ were easily maintained in the target range with both protocols (Table 1). Main acid-base parameters and phosphate during RCA-CVVH periods are reported, for both protocols, in Table 1 and Figure 2. Protocol A required bicarbonate (8.9±2.8 mmol/h) and sodium phosphate (5 g/d) infusion while protocol B allowed to stop both supplementations. Furthermore, the need for potassium supplementation was significantly lower with protocol B (Table 1). Hypomagnesemia has been prevented in both cases by magnesium sulfate supplementation (3 g/d) but serum magnesium was significantly higher with protocol B and at the lower reference limit during protocol A period (Table 1).

**Table 1:** RCA-CVVH settings, patient's main parameters and supplementation needs during the two treatment periods

RCA-CVVH settings		Protocol A	Protocol B
Blood flow rate (Qb) (mL/min)		140	140
Citrate flow rate (mL/h)		1680	900
Replacement solution flow rate (100% post) (mL/h)		600	1200
Fluid removal^a^ (mL/h)		100	100
Prescribed dialysis dose^b^ (mL/kg/h)		31.5	31.5

Patient's acid-base status, BUN, electrolytes, and supplementations during RCA-CVVH			

	Protocol A	Protocol B	P-value
pH (units)	7.39 ± 0.03	7.44 ± 0.03	<0.0001
Blood HCO_3_^−^ (mmol/L)	22.3 ± 1.8	22.6 ± 1.4	NS
Base excess (BE)	−2.8 ± 2.1	−1.6 ± 1.2	0.007
Blood urea nitrogen (BUN) (mg/dL)	34.8 ± 3.9	37.5 ± 1.2	NS
Systemic Ca^2+^ (mmol/L)	1.18 ± 0.06	1.15 ± 0.08	0.014
Circuit Ca^2+^ (mmol/L)	0.36 ± 0.04	0.36 ± 0.07	NS
Serum K^+^ (mmol/L)	4.0 ± 0.2	4.2 ± 0.3	NS
Serum Mg^2+^ (mmol/L)	0.73 ± 0.05	0.87 ± 0.07	0.001
Serum phosphate (mmol/L)	0.85 ± 0.2	1.3 ± 0.5	0.027
CaCl_2_ infusion (mmol/h)	2 ± 0.2	1.9 ± 0.5	NS
KCl infusion (mmol/h)	4 ± 0.2	1.4 ± 1.5	<0.0001
Magnesium sulfate (g/day)	3	3	–
Bicarbonate infusion (mmol/h)	8.9 ± 2.8	No supplementation	–
Phosphate supplementation (g/day)	5	No supplementation	–

Data are expressed as mean ± SD. Statistical comparison between two protocols: Student's *t*-test.

a Modified according to clinical needs.

b Corrected for predilution (correction factor = blood flow rate/[blood flow rate + predilution infusion rate]).

## Discussion

In our center, starting from June 2008, in high bleeding risk patients who underwent CRRT we have been adopting RCA as alternative to heparin or no anticoagulation. Until September 2011, a 12 mmol/L citrate solution, combined with a conventional postdilution replacement fluid with bicarbonate (32 mmol/L), has been used to perform RCA in CVVH modality (protocol A). This protocol provided an adequate RCA without electrolyte and/or metabolic derangements.^23^ Indeed, the use of a 12 mmol/L citrate solution allowed to maintain a low metabolic load, markedly reducing the risk of citrate accumulation. However, on the other side, the low amount of citrate delivered to the patient may be associated in many cases with a suboptimal buffers supply.^23^,^24^ As a consequence, despite optimization of CVVH parameters (i.e., citrate and/or postdilution bicarbonate solution flow rate), the persistence of a mild metabolic acidosis may require additional bicarbonate supplementation, as observed in this patient during protocol A period. Therefore, in our experience, if a higher buffer supply is needed, any increase in citrate flow rate with the use of a very low citrate concentration solution (12 mmol/L) results in a too slight rise in buffer supply to the patient. Comparable findings, regarding the need for additional bicarbonate, have been reported by Hetzel et al.,^24^ performing CVVH with a 13 mmol/L citrate solution, and by Shum et al.,^25^ adopting CVVH with a 12 mmol/L citrate solution combined with prefilter infusion of a highly concentrated bicarbonate solution (8.4%) to obtain a more positive buffers balance.

Our purpose, designing protocol B, was to evaluate the possibility to optimize buffers balance throughout the use of a more concentrated citrate solution (18 mmol/L) in combination with a new phosphate-containing replacement fluid with a standard bicarbonate concentration (30 mmol/L). Indeed, differently to protocol A, the higher concentration of citrate (18 mmol/L) could allow to significantly increase buffer supply throughout little increments of citrate flow rate.

The use of a 18 mmol/L citrate solution has been previously reported in CVVHDF modality by Tolwani et al..^26^ However, differently to our protocol B, the authors adopted a lower than usual dialysate bicarbonate concentration (25 mmol/L) to avoid alkalosis. In this preliminary single-patient experience, protocol B allowed to adequately control the acid-base status without additional bicarbonate infusion and in the absence of alkalosis, despite the use of a standard bicarbonate (30 mmol/L) replacement solution. In this regard, although these findings need to be confirmed in different patients and in a wider range of clinical situations, protocol B appears to represent a step forward if compared to protocol A. Indeed, if required, the protocol B provides room for additional buffer supply by increasing citrate flow rate. On the other hand, the handling of acid-base status may be more difficult in case of alkalosis. Indeed, the citrate dose is already low and any reduction in blood flow rate (to reduce citrate load) may be not appropriate or not easily applied in CVVH modality. Therefore, it should be recognized that the handling of acid-base status represents a potential limit of the protocol B in case of metabolic alkalosis.

In addition, protocol B allowed to reduce the amount of potassium chloride supplementation and to obtain more stable serum magnesium levels further reducing nursing workload. Furthermore, the combination of a phosphate-containing replacement fluid appeared effective to prevent hypophosphatemia, allowing to stop phosphate supplementation otherwise required during protocol A period. To our knowledge, this is the first report of the use of a calcium and phosphate-containing replacement solution during RCA. However, the adoption of a phosphate-containing solution is not new in CRRT. In this regard, it is well known that in critically ill patients, severe hypophosphatemia can cause generalized muscle weakness and even paralysis of the respiratory muscles, myocardial dysfunction, reduced peripheral vascular resistance and encephalopathy.^27^ Furthermore, Demirjian et al. reported that dialysis-induced hypophosphatemia was associated with a higher incidence of prolonged respiratory failure requiring tracheostomy.^18^ Therefore, in critically ill patients undergoing CRRT, it is suitable to correct hypophosphatemia by intravenous administration of phosphate or to prevent it by addition of phosphate to the replacement and/or dialysate solutions. To meet this target, the feasibility and safety of phosphate addition to conventional dialysate and replacement fluids have been successfully tested in adult and pediatric patients undergoing CRRT.^14^,^17^ More recently, in 14 patients undergoing CVVHDF, Broman et al. reported that the use of a commercially available phosphate-containing CRRT solution allowed to reduce the variability of serum phosphate and to prevent hypophosphatemia.^19^

Finally, citrate dose adopted in both described protocols is probably the lowest until now reported. On the other hand, is well known that the use of a higher citrate load might be potentially associated with an increased risk of citrate accumulation, especially in patients with marked hemodynamic instability and very high severity scores,^28^ frequently observed in the cardiac surgery ICU. We know that the use of a very low citrate dose could be associated with a filter life shorter than commonly reported with a conventional dose RCA. However, in this preliminary experience, target circuit-Ca++ (≤ 0.4 mmol/L) was achieved without further modification of citrate flow rate and the maintenance of the target was associated with an adequate circuit lifetime.

In conclusion, both protocols adopted in our center afforded RCA-CVVH simplification throughout the use of only two kinds of CRRT solutions. Moreover, although needing confirmation in an adequate number of patients, the new RCA-CVVH protocol B allowed to provide an appropriate buffers balance in absence of alkalosis, despite the use of an 18 mmol/L citrate solution combined to a standard concentration bicarbonate solution. Finally, the advantages related to the optimal acid-base control and to the prevention of hypophosphatemia, provided by protocol B, contribute to further reduce RCA complexity and to minimize the risk of errors related to bags and additional infusions handling.
